# An ecological study on the association between early childhood caries and intimate partner violence in 20 low- and middle-income countries: 2007-2017

**DOI:** 10.12688/aasopenres.13237.2

**Published:** 2021-12-16

**Authors:** Morenike Oluwatoyin Folayan, Mary O. Obiyan, Maha El Tantawi, Arthur Kemoli, Ola B. Al-Batayneh, Balgis Gaffar, Robert J. Schroth, Early Childhood Caries Advocacy Group

**Affiliations:** 1 Department of Child Dental Health, Obafemi Awolowo University, Ile-Ife, Nigeria, 22005, Nigeria; 2 Department of Demography, Faculty of Social Sciences, Obafemi Awolowo University, Ile-Ife, Nigeria, 22005, Nigeria; 3 Department of Pediatric Dentistry and Dental Public Health, Faculty of Dentistry, Alexandria University, Alexandria, Egypt; 4 Department Paediatric Dentistry and Orthodontics, University of Nairobi, Nairobi, Kenya; 5 Department of Preventive Dentistry, Faculty of Dentistry, Jordan University of Science and Technology, PO Box 3030, Irbid, 22110, Jordan; 6 Preventive Dental Sciences Department, College of Dentistry, Imam Abdulrahman bin Faisal University, Dammam, Saudi Arabia; 7 7Department of Preventive Dental Science, Dr. Gerald Niznick College of Dentistry, Rady Faculty of Health Sciences, University of Manitoba, Winnipeg, Manitoba, Canada

**Keywords:** Physical violence; Sexual violence; Emotional violence; Early childhood caries; Global; Gross National Income

## Abstract

**Background:** Women are the worst affected by intimate partner violence (IPV), and this impacts negatively on the health of the children they care for. This study aimed to determine the relationship between IPV and the prevalence of early childhood caries (ECC) in 3-5-year-olds.

**Methods:** This was an ecological study using IPV (physical, sexual, emotional) data extracted from the Demographic Health Survey of 20 low- and middle-income countries and ECC data for 3-5-year-olds of the same countries for the period 2007-2017. Linear regression analysis was used to assess the relationship between the percentage of 3-5-year-olds with ECC (outcome variable) and IPV indicators (physical, sexual, emotional). The model was adjusted for the country’s gross national income and the percentage of women with secondary or higher education. Partial eta squared (ηp ^2^), regression coefficients, confidence intervals and p-values were calculated.

**Results:** Data on ECC in 3-5-year-olds and IPV were available for six low-income-countries, 10 lower-middle-income-countries and four upper-middle-income-countries. The most prevalent form of IPV was physical violence (10.09%). The Democratic Republic of Congo had the highest prevalence of physical violence (45.8%), sexual violence (25.4%), and ECC (80.0%). The strongest association was between the prevalence of ECC and emotional violence (ηp ^2^=0.01), followed by physical violence (ηp ^2^=0.005), and sexual violence (ηp ^2^=0.003). For every 1% higher prevalence of emotional violence, there was 0.28% higher prevalence of ECC, and for every 1% higher percentage of physical violence, there was 0.21% higher prevalence of ECC. On the contrary, for every 1% higher prevalence of sexual violence, there was 0.35% lower prevalence of ECC prevalence.

**Conclusions:** The association between IPV and the prevalence of ECC in 3-5-year-olds is minor, with the strongest direct association effect being with emotional violence; and there was an inverse association between sexual violence and the prevalence of ECC. These findings need to be studied further.

## Introduction

Intimate partner physical, emotional and sexual violence (IPV) is a recognized threat to the health and rights of women globally ^1, 2^. Global estimates in 2010 indicated that about 30% of women have a history of IPV with this being highest in central (65.6%) and western (41.8%) sub-Saharan Africa and South Asia (41.7%) ^3^. IPV is defined as the abuse that takes place between a couple who are either married or living together and may or may not have a child ^4^. IPV not only has an impact on maternal health and welfare ^5^, It also affects the nutritional status of children of mothers affected by IPV: these children are more likely to be stunted ^6– 12^. In addition, children who are exposed to IPV have increased risk of behavioural, emotional, psychological and social problems including mood and anxiety disorders, post-traumatic stress disorder, substance abuse and school-related problems ^13^. Abused women are also more likely to physically abuse their children ^14– 16^. Children of mothers who experience IPV are also less likely to be immunized; have higher rates of diarrhoeal disease; and/or are at greater risk of dying before the age of five ^17, 18^

The negative impact on the mental health of women affected by IPV ^19^ results in poor nutritional care of children ^20– 25^ causing an increased risk for stunting ^6– 10, 12^, and poor or delayed medical care access by affected children ^12, 23– 25^. In addition, women who experience IPV may resort to substance use as a way to cope with the emotional, physical and mental stress ^26^. Substance use may also be a way for affected persons to maintain and/or establish intimate relations with others ^27^. Substance use disorder is a risk factors for child abuse and poor management of the oral health of the child. It may affect a parent’s ability to function as a caregiver and provide basic health care needs of the child ^28^.

Though several studies have established the negative association between maternal mental health and increased risk for ECC ^29– 32^, there is little known about the association between IPV and ECC. Lorber *et al.* ^33^ studied children’s exposure to domestic violence and the risk for ECC and found no significant relationship between them though a positive trend was observed. However, in households where women were aggressive towards men, the risk for ECC was higher due to high exposure to cariogenic diets ^34^.

The social cognitive theory may explain the relationship between IPV and ECC. The theory recognizes that an individual’s behavior, cognition, and environment reciprocally and dynamically influence one another ^35^. Key social environmental and personal factors play roles in determining an individual’s engagement in health-promoting and disease-preventing behaviors ^36^. Self-efficacy is a central cognitive construct in the social cognitive theory that may predict and explain health behaviors and outcomes ^37– 39^. Thus, a person’s belief or confidence in their ability to perform certain actions influences the decisions to perform these actions ^40^, including tooth brushing ^41^, which is a significant risk factor for poor oral hygiene and ECC ^42^. IPV undermines the self-efficacy of affected individuals ^43, 44^ who are mostly women ^45^ serving as caretakers for children ^46^. IPV can therefore compromise the ability of women to care for children who are entirely dependent on them for daily oral hygiene and access to professional oral health care services.

The aim of this study was to determine the relationship between the country-level prevalence of IPV and the prevalence of ECC in children aged 3-5-year-olds in countries where data on IPV and ECC are available. We conducted a country-specific analysis given the evidence suggesting differences in the relationship between IPV and ECC ^47^.

## Methods

### Study design and data sources

This ecological study was based on IPV data extracted from the domestic violence module of the Demographic Health Survey (DHS), a nationally representative household survey that collects information on population-based indicators of health and nutrition in low- and middle-income countries (LMICs). The module includes questions on types and experiences of violence (physical, sexual and emotional) mostly perpetrated by the current or most recent husband/ partner ^48^. 

The present study also used data on ECC collected by El Tantawi *et al.* ^49^. The data on the prevalence of ECC between 2007 and 2017 was extracted from the WHO and other Country Oral Health Profile databases as well as from published journals and government reports after conducting a search applying no language filter. Data on ECC was generated for 113 countries using this methodology.

The Data for both IPV and ECC between 2007 and 2017 were available in only 20 LMICs (Cambodia, Columbia, Democratic Republic of Congo, Egypt, Gambia, India, Kenya, Kyrgyzstan, Myanmar, Namibia, Nepal, Nigeria, Pakistan, Peru, Philippines, Senegal, South Africa, Tanzania, Uganda and Ukraine).

### Early childhood caries

ECC is identified in children < 72 months of age with one or more decayed (cavitated and non-cavitated), missing teeth due to decay or filled primary tooth surfaces ^50^. For each country, databases were searched for data on ECC prevalence: (1) MEDLINE, (2) Web of Science (3) Scopus and (4) Google Scholar (1 ^st^ 100 search results) using a country’s name combined with search terms. This was complemented by searching master and doctoral degrees theses about ECC, articles published in local journals and government reports. No language filter was applied.

Studies were included if they (1) were conducted on humans in epidemiologic, non-laboratory setting, (2) assessed ECC using the definition of the American Academy of Pediatric Dentistry or data was available to allow its estimation and (3) included healthy children. Excluded studies were (1) duplicates such as theses and resulting publications, (2) publications from which needed information could not be extracted (article with no full text or information not mentioned) and (3) studies published before 2007.

We extracted data from studies reporting prevalence figures from nationally representative samples if they were available. Otherwise, we used the retrieved data to calculate ECC prevalence for a country by dividing the total number of children affected by ECC in the study/s by the total number of children examined and multiplying by100.

The search strategy for Pubmed was: "Dental Caries"[Mesh] OR caries OR dental decay; Filter by age “infant: 1–23 months” and “preschool child”; Filter by date: from January 1 ^st^ 2007 to December 31 ^st^ 2017; Combine with “Country x” [Mesh] using “AND” The search term was as follows in case of Egypt for example: (("Dental Caries"[MeSH Terms] OR ("carie"[All Fields] OR "Dental Caries"[MeSH Terms] OR ("dental"[All Fields] AND "caries"[All Fields]) OR "Dental Caries"[All Fields] OR "caries"[All Fields]) OR ("Dental Caries"[MeSH Terms] OR ("dental"[All Fields] AND "caries"[All Fields]) OR "Dental Caries"[All Fields] OR ("dental"[All Fields] AND "decay"[All Fields]) OR "dental decay"[All Fields])) AND "Egypt"[MeSH Terms]) AND ((2007:2017[pdat]) AND (infant[Filter] OR preschool child [Filter])).

No language filter was applied. The retrieved data were used to calculate the ECC prevalence for each country by dividing the number of children affected by ECC by the number of children examined and multiplying by 100. In the present study, we used the prevalence of ECC for 3–5-year-old children. Further details were reported in our previous paper ^49^.

### Intimate partner violence

We investigated the history of physical violence (the intentional use of physical force with the potential to cause injury or harm), sexual violence (any experience of unwanted or forced sexual activity and sexual coercion) and emotional violence (psychological abuse including humiliation, insults and intimidation) ^51^. Self-reported lifetime exposure to IPV was separated into three categories: (i) physical violence only; (ii) sexual violence only; and (iii) emotional violence only. Details on the source of the IPV is available in Table 1.

**Table 1.** Country Demographic Health Survey (DHS) and URL.

COUNTRY	DHS YEAR OF SURVEY	DATA URL
Cambodia	2014	https://dhsprogram.com/customcf/legacy/data/download_dataset.cfm?Filename=KHIR73DT.ZIP&Tp=1&Ctry_ Code=KH&surv_id=464&dmode=normal
Colombia	2015	https://dhsprogram.com/customcf/legacy/data/download_dataset.cfm?Filename=COIR72DT.ZIP&Tp=1&Ctry_ Code=CO&surv_id=476&dmode=normal
Congo, DRC	2013/14	https://dhsprogram.com/customcf/legacy/data/download_dataset.cfm?Filename=CDIR61DT.ZIP&Tp=1&Ctry_ Code=CD&surv_id=421&dmode=normal
Egypt	2014	https://dhsprogram.com/customcf/legacy/data/download_dataset.cfm?Filename=EGIR61DT.ZIP&Tp=1&Ctry_ Code=EG&surv_id=397&dmode=normal
Gambia	2013	https://dhsprogram.com/customcf/legacy/data/download_dataset.cfm?Filename=GMIR61DT.ZIP&Tp=1&Ctry_ Code=GM&surv_id=425&dmode=normal
India	2015/16	https://dhsprogram.com/customcf/legacy/data/download_dataset.cfm?Filename=IAIR74DT.ZIP&Tp=1&Ctry_ Code=IA&surv_id=355&dmode=cf
Kenya	2014	https://dhsprogram.com/customcf/legacy/data/download_dataset.cfm?Filename=KEIR72DT.ZIP&Tp=1&Ctry_ Code=KE&surv_id=451&dmode=normal
Kyrgyzstan	2012	https://dhsprogram.com/customcf/legacy/data/download_dataset.cfm?Filename=KYIR61DT.ZIP&Tp=1&Ctry_ Code=KY&surv_id=383&dmode=normal
Myanmar	2015/16	https://dhsprogram.com/customcf/legacy/data/download_dataset.cfm?Filename=MMIR71DT.ZIP&Tp=1&Ctry_ Code=MM&surv_id=454&dmode=normal
Namibia	2013	https://dhsprogram.com/customcf/legacy/data/download_dataset.cfm?Filename=NMIR61DT.ZIP&Tp=1&Ctry_ Code=NM&surv_id=363&dmode=normal
Nepal	2016	https://dhsprogram.com/customcf/legacy/data/download_dataset.cfm?Filename=NPIR7HDT.ZIP&Tp=1&Ctry_ Code=NP&surv_id=472&dmode=normal
Nigeria	2013	https://dhsprogram.com/customcf/legacy/data/download_dataset.cfm?Filename=NGIR6ADT.ZIP&Tp=1&Ctry_ Code=NG&surv_id=438&dmode=normal
Pakistan	2017/18	https://dhsprogram.com/customcf/legacy/data/download_dataset.cfm?Filename=PKIR71DT.ZIP&Tp=1&Ctry_ Code=PK&surv_id=523&dmode=normal
Peru	2012	https://dhsprogram.com/customcf/legacy/data/download_dataset.cfm?Filename=PEIR6IDT.ZIP&Tp=1&Ctry_ Code=PE&surv_id=434&dmode=normal
Philippines	2017	https://dhsprogram.com/customcf/legacy/data/download_dataset.cfm?Filename=PHIR71DT.ZIP&Tp=1&Ctry_ Code=PH&surv_id=510&dmode=normal
Senegal	2017	https://dhsprogram.com/customcf/legacy/data/download_dataset.cfm?Filename=SNIR7ZDT.ZIP&Tp=1&Ctry_ Code=SN&surv_id=534&dmode=normal
South Africa	2016	https://dhsprogram.com/customcf/legacy/data/download_dataset.cfm?Filename=ZAIR71DT.ZIP&Tp=1&Ctry_ Code=ZA&surv_id=390&dmode=normal
Tanzania	2015/16	https://dhsprogram.com/customcf/legacy/data/download_dataset.cfm?Filename=TZIR7BDT.ZIP&Tp=1&Ctry_ Code=TZ&surv_id=485&dmode=normal
Uganda	2016	https://dhsprogram.com/customcf/legacy/data/download_dataset.cfm?Filename=UGIR7BDT.ZIP&Tp=1&Ctry_ Code=UG&surv_id=504&dmode=normal
Ukraine	2007	https://dhsprogram.com/customcf/legacy/data/download_dataset.cfm?Filename=UAIR51DT.ZIP&Tp=1&Ctry_ Code=UA&surv_id=280&dmode=normal

Demographic Health Survey data are publicly available at: https://dhsprogram.com/

### Confounders

Income level was reported to be associated with ECC ^49^ and IPV ^3^. We adjusted for country income level based on the 2015 gross national income (GNI) per capita calculated using the World Bank Atlas method ^52^ since it was the latest available for the study period. In addition, we also obtained information about the percentage of women between 15 and 49 years of age who completed secondary or higher education per country from the DHS data ^53^.

### Statistical analysis

The datasets (ECC and IPV indicators) were matched by country. Analysis was restricted to only women selected and successfully interviewed in the DHS domestic module and those who were formerly or currently in union or living with a man at the time of survey. Statistics, including means and standard deviations (SD), were calculated for IPV indicators, ECC, GNI and percentage of women completing secondary or higher education. Multiple linear regression analysis was used to assess the relationship between the percentage of 3-5-year-old children with ECC (outcome variable) and the three IPV indicators adjusted for GNI and percentage of women with secondary or higher education. Regression coefficients, 95% confidence intervals (CIs), p values and partial eta squared (η _p_^2^) as the measure of effect size were calculated using SPSS version 22.0 (IBM Corp., Armonk, N.Y., USA). Significance level was set at 5%.

## Results

A total of 190 publications on ECC from 88 countries (45.6% of the UN member states) were available ^54^. Of these, data on ECC in 3-5-year-olds and IPV were available for 20 LMICs. These 20 countries included six low-income countries (30%), 10 lower-middle-income countries (50%) and four upper-middle-income countries (20%). In view of the small number of countries with available data, analysis was conducted for all 20 countries collectively rather than categorizing by income level. In total 68,031 children <36 months old and 154,452 children 36–71 months old were included in the study. 

Table 2 lists the GNI, percentage of women with secondary or higher education, the percentage of 3-5-year-old children with ECC, and the proportion of women in the country who reported (i) physical violence only; (ii) sexual violence only; and (iii) emotional violence only. The prevalence (SD) of ECC in 3-5-year-olds in these 20 countries was 62.20% (18.66) and the mean (SD) GNI was 6219.47 (4083.99) US$ whereas the mean (SD) percentage of women with secondary or higher education was 57.15% (23.47). The most reported form of IPV was physical violence (25.46%, 10.09), followed by emotional violence (22.19%, 9.90), and lastly sexual violence (7.87%, 6.18).

**Table 2.** Distribution of early childhood caries (ECC) and indicators of interpersonal violence in the 20 countries included in the study. SD=standard deviation.

ID	Country	Gross national income in US dollars	Percentage of women with secondary or higher education	ECC prevalence in children aged 3–5- year-old %	Number of survey participants n	Country level prevalence of physical violence n (%)	Country level prevalence of sexual violence n (%)	Country level prevalence of emotional violence n (%)
1	Cambodia	3300	40.1	78.8	3,499	536 (15.3%)	164 (4.7%)	790 (22.6%)
2	Colombia	13550	82.7	75.3	24,862	8,384 (33.7%)	1,985 (8.0%)	---
3	Congo, DRC	720	47.7	80.0	5,691	2,606 (45.8%)	1,448 (25.4%)	2,073 (36.4%)
4	Egypt	10710	65.7	61.6	6,693	1,590 (23.8%)	267 (4.0%)	1,238 (18.5%)
5	Gambia	-	39.8	86.0	3,542	784 (22.1%)	103 (2.9%)	592 (16.7%)
6	India	6030	60.1	52.1	66,013	18,680 (28.3%)	4,372 (6.6%)	8.372 (12.7%)
7	Kenya	3070	46.2	64.2	4,519	1,609 (35.6%)	529 (11.7%)	1,373 (30.4%)
8	Kyrgyzstan	3310	99.5	69.8	4,832	1,283 (26.6%)	201 (4.2%)	592 (12.3%)
9	Myanmar	4930	46.3	50.0	3,425	575 (16.8%)	131 (3.8%)	544 (15.9%)
10	Namibia	10380	75.8	68.7	1,449	359 (24.8%)	106 (7.3%)	367 (25.3%)
11	Nepal	2500	50	61.5	3,826	842 (22.0%)	297 (7.8%)	485 (12.7%)
12	Nigeria	5810	50.6	14.9	22,305	3,385 (15.2%)	1,190 (5.3%)	4,477 (20.1%)
13	Pakistan	5320	34.3	60.0	4,085	1,004 (24.6%)	184 (4.5%)	1,243 (30.4%)
14	Peru	12060	75.2	76.0	13,483	5,063 (37.6%)	1,235 (9.2%)	4,187 (31.1%)
15	Philippines	8940	86.3	93.0	13,215	1,486 (11.2%)	591 (4.5%)	2,903 (22.0%)
16	Senegal	2380	32.3	73.0	2,660	546 (20.5%)	167 (6.3%)	357 (13.4%)
17	South Africa	12870	88.9	44.0	2,354	356 (15.1%)	90 (3.8%)	451 (19.2%)
18	Tanzania	2630	22.9	37.3	15,194	5,512 (36.3%)	1,882 (12.4%)	4,904 (32.3%)
19	Uganda	1820	36.1	41.0	7,536	3,138 (41.6%)	1,710 (22.7%)	3,141 (41.7%)
20	Ukraine	7840	99.9	56.7	2,453	304 (12.4%)	59 (2.4%)	510 (20.8%)
	Mean (SD)	6219.47 (4083.99)	57.15 (23.47)	62.20 (18.66)	10,581 (14,6667)	25.46 (10.09%)	7.87 (6.18%)	22.19 (9.90%)

The Democratic Republic of Congo (DRC), a war-torn country, had the highest prevalence of physical violence (45.8%). ECC prevalence in this country was also high (80%). Sexual violence was highest in DRC, followed by Uganda (25.4%). The highest prevalence of emotional violence (41.7%) was in Uganda while the prevalence of ECC in Uganda was 41%.

Table 3 highlights the association between the percentage of 3-5-year-old children with ECC and the percentage of women with various types of IPV in the 20 LMICs after adjusting for GNI and the percentage of women with secondary and higher education. The associations between ECC and each of the three categories of IPV were non-statistically significant. The strongest association was between ECC prevalence and exposure to emotional violence (ηp2= 0.01), followed by exposure to physical violence (ηp2= 0.005), and exposure to sexual violence (ηp2= 0.003). Multiple linear regression analysis revealed that for every 1% higher percentage of women reporting exposure to physical violence, there was a 0.21% non-statistically significantly higher prevalence of ECC. For every 1% higher prevalence of sexual violence, countries had 0.35% non-statistically significantly lower ECC prevalence while for every 1% higher prevalence of emotional violence, countries had non-statistically significant 0.28% higher ECC.

**Table 3.** Association between the percentage of 3–5-year-old children with early childhood caries (ECC) and the percentage of women with various types of interpersonal violence (IPV) in 20 low- and middle-income countries (LMICs).

Factors	Regression coefficient (95% CI)	P value	Partial eta squared (ηp ^2^)
Prevalence of physical violence	0.21 (-1.74, 2.17)	0.82	0.005
Prevalence of sexual violence	-0.35 (-4.33, 3.62)	0.85	0.003
Prevalence of emotional violence	0.28 (-1.27, 1.83)	0.70	0.01

Adjusted for country gross national income (GNI) and percentage of women with secondary or higher education, CI: confidence interval

## Discussion

This is the first study attempting to assess the association between IPV and ECC using national data available from multiple countries around the world. This data suggests that in countries with a higher proportion of women facing physical and emotional IPV, the prevalence of ECC is also higher. However, the prevalence of ECC was lower in countries with a higher proportion of women facing sexual IPV. The relationships between the forms of IPV and ECC examined in this study were not statistically significant likely due to the relatively few numbers of countries with available information from the DHS. Also, the effect sizes observed were small though this is not unexpected given the multifactorial nature of ECC, and that IPV may be partly explained by other social determinants of health such as income and education (for which we controlled for in our analysis). The study finding, however, highlights an area for future research as there are indications that the global incidence of IPV has been on the increase ^55^; and the association between both IPV and ECC seems to represent co-existing issues rather a causal relationship. Factors such as culture, lifestyle and socio-economic status may be etiological or predisposing factors for ECC and IPV. Although we controlled for income and education, other related underlying factors that are not measured may be able to explain some of the observed variance among countries in the prevalence of both health problems (IPV and ECC).

The study provides new information to strengthen existing evidence on how social context is associated with disease risks. Globally, the prevalence of ECC is high – it is the 10 ^th^ most prevalent childhood illness ^56^. Most of the efforts to address this problem have largely been limited to interventions aiming at altering biological and behavioral risk factors. Little attention has been paid to addressing the social determinants of health, significantly impact the behavioral and biological risks.

The study results suggest that the children 3-5-years-old resident in LMICs may be at increased risk for ECC when the care-giver is exposed to IPV though the effect size may be small. The stronger link between emotional violence and ECC – stronger than physical and sexual violence –may be related to post-traumatic stress disorder ^57^, suicidal ideation and low self-esteem ^58^ that results from the emotional violence. Emotional abuse often occurs prior to or concurrently with physical or sexual abuse and increases the trauma of physical and sexual abuse ^59^. The risk for mental health consequences of emotional violence is higher than it is for physical and sexual violence ^58^. Children of mothers with mental health disorders are more likely to have ECC than children of mothers without these mental health disorders ^60^.

The non-significant associations between ECC and the different types of IPV and the small effect size observed may imply that the role of IPV as a structural determinant of the risk for ECC may be small for the children at this age. However, with our understanding that when children 3-5-years-old are exposed to IPV as an adverse childhood event, the risk of caries in adolescence and adulthood is significantly higher ^61^. What this study has done is to indicate that there are signals of risk for caries when pre-schoolers are exposed to IPV. Existing studies and reviews of the IPV and oral health literature reveal that the link between IPV and ECC is quite plausible ^62^. Integrating early childhood promotion and ECC prevention messages into programs that take place in women’s shelters may be the first step to reduce the immediate possible risk of ECC and possible future risk for caries by offering practical methods to women to care for their children’s oral health. This proposed management strategy also reiterates the importance of integrated maternal and child health care as a cost-effective strategy to manage diseases using the common risk approach ^63^. There is currently very little evidence on approaches that promote the integration of maternal and child general healthcare with their – and especially the child’s- oral health. As evidence emerges, it becomes needful to develop programs addressing maternal IPV and ECC in pre-school children as part of a general health screening process, especially in countries where the prevalence of IPV is high.

The inverse though non-significant relationship between sexual violence and the prevalence of ECC is difficult to explain. Studies suggest that some mothers who had experienced sexual abuse demonstrate parental reflective functioning, an essential capacity for providing sensitive care for children under routine parenting circumstances. This is also associated with the caregiver developing resilience ^64, 65^. This evidence of increased child protection by mothers who have a history of sexual violence may also be reflected in the improved oral healthcare the children receive. There is however, research that demonstrated negative psychosocial impact of sexual violence with negative implications for the oral health of children ^66^ while another found no association between women’s experience of violence and ECC ^67^. This finding needs to be studied further.

One of the strengths of the present study is the use of population-level data derived from the DHS, thus ensuring data quality ^68^. We used the data from 20 LMICs to provide insight into a phenomenon that if further explored, may shape policy and practice around ECC management ^69^. Our study, however, is limited by the low number of included countries thereby challenging the generalizability of findings. The low sample size was addressed by using partial eta squared, a measure of effect size, to compare the association of ECC with various forms of IPV and to decide on the importance of each variable, rather than using the p values to determine the significance of the associations. Ensuring the availability of IPV data in other countries with different income levels may help develop a better understanding of the nature of the association between ECC and IPV.

Another limitation is that IPV data is based on self-reporting with the possibility of under-reporting due to social desirability bias ^70^. However, this is the standard method used to obtain information on IPV ^71^. We also only extracted data on IPV and women though there is evidence suggestive that both males and females may be victims of IPV ^72, 73^; and IPV affecting men may be associated with the risk of ECC ^26^. Also, this is a cross-sectional study. We are unable to determine a causal relationship between IPV and ECC and the direction of the relationship between the two variables though it seems less likely that ECC will cause IPV. In addition, the study is ecological with potential for ecological fallacy resulting from linking different independent data sets ^74, 75^. The 20 countries included in this study were not randomly selected to represent the 135 LMICs ^76^. Rather they were a sample of LMICs with complete data for the variables included in this study. This limits the generalizability of the study findings to all LMICs. However, the report can be generalized to countries with similar epidemiological profiles to those represented in this study. Like all ecological studies, our study suggests the plausibility of an association between ECC and IPV although the effect size in the present case is small. Future studies are needed to look into possible associations at the individual level and assess the presence of potential mediating factors.

## Conclusions

Despite these limitations, the study provides new insight into the possible association between ECC and IPV: physical, sexual and emotional violence were associated with ECC prevalence though the associations were not significant, and the effect size of the associations were small. Women’s exposure to emotional violence had stronger associations with the prevalence of ECC than physical and sexual violence.

## Data availability

### Underlying data

The IPV data used in this study are available from the Demographic and Health Survey (DHS) website. Access to the dataset requires registration and is granted only for legitimate research purposes. A guide for how to apply for dataset access is available at: https://dhsprogram.com/data/Access-Instructions.cfm. Please find the urls to the specific datasets in Table 1.

### Extended data

Figshare: supplementary files for ECC and IPV manuscript. https://doi.org/10.6084/m9.figshare.15000324.v1 ^54^.

This project contains the following extended data:

- supplementary file_IPV.doc (List of studies reporting early childhood caries prevalence)
- IPV_ECC.do (Analysis code)

Data are available under the terms of the Creative Commons Attribution 4.0 International license (CC-BY 4.0).
