# Supplementary material for: An ecological study on the association between early childhood caries and intimate partner violence in 20 low- and middle-income countries: 2007-2017
Source: AAS Open Res. 2022 Feb 18;4:39. Originally published 2021 Jul 26. [Version 3] doi: 10.12688/aasopenres.13237.3 (PMC9648360; doi:10.12688/aasopenres.13237.3)
Supplement: Peer review history

# Peer review: Reviewer response for version 3

**DOI:** 10.21956/aasopenres.14480.r29161

**Published:** 2022-04-06

**Authors:** Mohajer Abbass Hameed

**Affiliations:** 1 Murdoch Children's Research Institute, Melbourne, Vic, Australia

This study utilises a cross-sectional methodology to explore the association between IPV and ECC using national data (Demographic and Health Survey) available from multiple countries. Overall, whilst the findings are statistically not significant, the authors, on some occasions (e.g. in discussion) appear to over-emphasise the findings and may appear to indicate that there is an association. Nevertheless, the key message that the findings do not support the hypothesis for a significant association, is sufficiently articulated.

 In the method section, the authors indicate that IPV data was extracted from the domestic violence module of the Demographic Health Survey and authors have provided relevant data websites. However, in terms of prevalence of ECC, the authors appear to have done a literature search and identified articles/publications; yet it is not clear as to whether this was a systematic search of the selected databases, and how screening of records were done. Or was this a rapid search of the literature to get the relevant articles, if so, how many articles were included/excluded? Furthermore, when IPV data is extracted from DHS and ECC from published papers, how were these linked together? In other words, IPV data is extracted from one source (DHS) and ECC data extracted from another source (literature search); then how were these merged or analysed together? It appears that there is some missing sentence/information in the method section to clarify this.

 Throughout the manuscript, there are some typos and grammatical errors. For example punctuation needs to be corrected in page 4, “IPV not only has an impact on maternal health and welfare ^5^, It also affects the nutritional status of children of mothers affected by IPV: these children are more likely to be stunted ^6–12^.”

 Lastly, the title of the manuscript can be re-worded to reflect the study investigation. Currently, the title indicates “association between early childhood caries and intimate partner violence”, I wonder if this should be reversed to “association between IPV and ECC".

Is the work clearly and accurately presented and does it cite the current literature?

Yes

If applicable, is the statistical analysis and its interpretation appropriate?

Yes

Are all the source data underlying the results available to ensure full reproducibility?

Yes

Is the study design appropriate and is the work technically sound?

Partly

Are the conclusions drawn adequately supported by the results?

Yes

Are sufficient details of methods and analysis provided to allow replication by others?

Partly

Reviewer Expertise:

Developmental psychology, intergenerational and family health, statistics,

I confirm that I have read this submission and believe that I have an appropriate level of expertise to confirm that it is of an acceptable scientific standard.

# Peer review: Reviewer response for version 3

**DOI:** 10.21956/aasopenres.14480.r29131

**Published:** 2022-03-04

**Authors:** Shirin Ziaei

**Affiliations:** 1 International Maternal and Child Health Unit (IMCH), Department of Women's and Children's Health, University Hospital, Uppsala University, Uppsala, Sweden

I thank authors for their great efforts in improving the paper and and addressing the comments.

Is the work clearly and accurately presented and does it cite the current literature?

Yes

If applicable, is the statistical analysis and its interpretation appropriate?

I cannot comment. A qualified statistician is required.

Are all the source data underlying the results available to ensure full reproducibility?

Yes

Is the study design appropriate and is the work technically sound?

Partly

Are the conclusions drawn adequately supported by the results?

No

Are sufficient details of methods and analysis provided to allow replication by others?

Partly

Reviewer Expertise:

Intimate partner violence_Maternal and child health_epidemiology

I confirm that I have read this submission and believe that I have an appropriate level of expertise to confirm that it is of an acceptable scientific standard.

# Peer review: Reviewer response for version 2

**DOI:** 10.21956/aasopenres.14454.r29064

**Published:** 2021-12-21

**Authors:** Shirin Ziaei

**Affiliations:** 1 International Maternal and Child Health Unit (IMCH), Department of Women's and Children's Health, University Hospital, Uppsala University, Uppsala, Sweden

Thank you so much for the new version of the paper. Several of the comments have been answered. The paper is well written and clear. However, I still think that the interpretation of the results and regression analysis is misleading and the fact that the p values are very large indicates that the findings might be by chance. So, although the authors mentioned that the findings are “insignificant” still the whole result section, discussion, and also abstract are written in a form that points to the existence of association rather than non-existence. I understand that the sample size is small but with the current sample size and p values, it is not possible to draw such conclusions. Further, the Cis were not adjusted for the cluster sampling design which is also a major issue to consider.

**Some minor details:**

- Please change the term “relationship” to “association” throughout the manuscript. The study is cross-sectional, and causality cannot be established.

- It is helpful if the authors can mention what are the criteria of ECC from the American Academy of pediatric dentistry.
- I still have a problem with including children below 72 months old. That means the included children were under 6 yrs (full 6 years) not five and what about the criteria of age above 3 years? If the authors only restricted the search to the children under 72 months that means that children younger than 3yrs are also included. Also in the result section, it has been mentioned 68031 children were below 36 months. That is in contradiction with the method section and aim that mentioned children 3-5 years are studied.
- Please clarify the variables for the experience of IPV, the authors mentioned that they have 3 variables for IPV: “physical violence only” “sexual violence only” and “emotional violence only” does that mean that if the women experienced both physical and sexual violence for example they are not included? Violence is hardly a single act and usually, the women experience multiple forms of violence. Perhaps the authors meant the experience of “any physical violence” etc. rather than “physical violence only”
- It is great that the authors used the weight for their statistical analysis but that is not enough when it comes to the p value and CI. They need to adjust for cluster sampling design by using the strata and cluster variables in the complex sample analysis. The current analysis is actually prone to type one error and the current p values after adjustment for the sampling design might be even bigger.

Is the work clearly and accurately presented and does it cite the current literature?

Yes

If applicable, is the statistical analysis and its interpretation appropriate?

I cannot comment. A qualified statistician is required.

Are all the source data underlying the results available to ensure full reproducibility?

Yes

Is the study design appropriate and is the work technically sound?

Partly

Are the conclusions drawn adequately supported by the results?

No

Are sufficient details of methods and analysis provided to allow replication by others?

Partly

Reviewer Expertise:

Intimate partner violence_Maternal and child health_epidemiology

I confirm that I have read this submission and believe that I have an appropriate level of expertise to state that I do not consider it to be of an acceptable scientific standard, for reasons outlined above.

# Response

**Published:** 2022-01-04

**Authors:** Morenike Oluwatoyin Folayan

**Affiliations:** Obafemi Awolowo University, Nigeria

**However, I still think that the interpretation of the results and regression analysis is misleading and the fact that the p values are very large indicates that the findings might be by chance. So, although the authors mentioned that the findings are “insignificant” still the whole result section, discussion, and also abstract are written in a form that points to the existence of association rather than non-existence. I understand that the sample size is small but with the current sample size and p values, it is not possible to draw such conclusions. Further, the Cis were not adjusted for the cluster sampling design which is also a major issue to consider.**

- Response: We understand and acknowledge the Reviewer’s concern. Our study is ecological as indicated at the beginning of Methods. This means the units of observation and analysis are countries not persons. Because of this, our sample size is 20. This is very much under-powered to show significance. Statistical significance, however, is a function of sample size. Please check the following table. We replicated our data (n=20) several times using the same values so that the sample would reach a much greater number (n= 1440) and re-ran the same analysis. In the re-ran analysis with a greater sample size of 1440, the regression coefficients (B) and partial eta squared are exactly the same as that of our sample of 20 but the confidence intervals changed and the p values are all significant.  

Prevalence of physical violence

 B = 0.21; p value = 0.01; 95% CI: 0.04-0.38; Partial Eta Squared = 0.005

 Prevalence of sexual violence

 B = - 0.35; p value = 0.045; 95% CI: -0.70 -  -0.008; Partial Eta Squared = 0.003

 Prevalence of emotional violence

 B = ​​​​​​​- 0.28; p value = <0.001; 95% CI: 0.15 -  0.42; Partial Eta Squared = 0.013

 Our study used an ecological design where the countries were the units of observation. If the same datasets are used and the analysis is re-run at person level, the sample size would be 154,452 instead of 20 and the significance would be even greater.

 Because of this, we include the effect size (partial eta squared) and considered it safe to mention the presence of associations rather than their absence. We made sure that we clarified all over the paper than the association was not significant and included the data so that our results can be replicated. We contend that there are always associations and the analysis can show whether they are significant or not (based on sample size among other things) and can also show their strength (for which we calculated the partial eta squared). 

 We feel our reporting and discussions of the associations and effect size were very appropriate for this form of data analysis. We have however gone ahead to revise the manuscript to focus on the non-significant association observed

**It is great that the authors used the weight for their statistical analysis but that is not enough when it comes to the p value and CI. They need to adjust for cluster sampling design by using the strata and cluster variables in the complex sample analysis. The current analysis is actually prone to type one error and the current p values after adjustment for the sampling design might be even bigger.**

- Response: Because we used ecological design, we calculated means for all values at country level. If the same datasets are used so that the person is the unit of analysis, persons would be clustered in countries and multilevel analysis would be needed to account for the clustering. Please check our response above clarifying that in our design where the unit of analysis is the country not the person, the values used are means of country values not persons and therefore there is no clustering and no multilevel analysis is needed.

**Some minor details: Please change the term “relationship” to “association” throughout the manuscript. The study is cross-sectional, and causality cannot be established. **

- Response: All the terms “relationship” changed to “association”

○ **It is helpful if the authors can mention what are the criteria of ECC from the American Academy of pediatric dentistry**.

- Response: Reference 50 defines Early childhood caries. The definitions indicate that the criteria for ECC is caries in children below 72 months old. ECC is an age based definition for caries. There are no other criterion for its definition. Other criteria are related to the determination of a diagnosis of caries.

○ **I still have a problem with including children below 72 months old. That means the included children were under 6 yrs (full 6 years) not five and what about the criteria of age above 3 years? If the authors only restricted the search to the children under 72 months that means that children younger than 3yrs are also included. Also in the result section, it has been mentioned 68031 children were below 36 months. That is in contradiction with the method section and aim that mentioned children 3-5 years are studied.**

- Response: We have edited this to 36 – 71 months. Also, the data of children below 36 months was not included in the analysis. This was an error and we have edited this. Thanks for picking this up.

○ **Please clarify the variables for the experience of IPV, the authors mentioned that they have 3 variables for IPV: “physical violence only” “sexual violence only” and “emotional violence only” does that mean that if the women experienced both physical and sexual violence for example they are not included? Violence is hardly a single act and usually, the women experience multiple forms of violence. Perhaps the authors meant the experience of “any physical violence” etc. rather than “physical violence only” **

- Response: The DHS data used series of items/questions to capture the form of violence experienced - physical, sexual, emotional. Women who answered positive to a particular item/question are categorized to have experienced the form of violence. While it is possible to generate a variable for women who experienced multiple forms of violence, this study however presents only an experience of each form of violence. We have therefore revised the variable in line with the suggestion and we have included this as a study limitation.

# Peer review: Reviewer response for version 1

**DOI:** 10.21956/aasopenres.14358.r28950

**Published:** 2021-11-01

**Authors:** Shirin Ziaei

**Affiliations:** 1 International Maternal and Child Health Unit (IMCH), Department of Women's and Children's Health, University Hospital, Uppsala University, Uppsala, Sweden

The paper discussed a very important issue and has a very interesting and important research question. However, I am concerned that the interpretation of the results is misleading, and the authors have gone a bit too far in trying to interpret the non-significant findings. The results as I can see are that there is no association between maternal experience of IPV and ECC. Which, as the authors mentioned, might be because of a small sample size. However, based on the p values and effects size the interpretation cannot go beyond. Please find some detailed comments below:

 Introduction:

- I believe the author can develop on the association between maternal experience of IPV and child health. While stunting is definitely one of the measured outcomes, there are several negative outcomes on children’s health which can be mentioned. Further, the pathways through which maternal experience of IPV can affect their children’s health can be discussed a bit more. The authors have a very strong approach by using social cognitive theory. As much as self-efficacy is an important and very well-known reason, there are other possible explanations linking maternal exposure to violence and their children’s health including dental health.
- “Countries where data is available” is quite a vague description. I recommend that authors talk about the number of the countries, region and list of them.

Methods.

- More information regarding DHS and El tantawi study is required.
- The number of included and excluded studies is needed in the methods.

Statistical analysis:

- I am not an expert in ecological studies but I know in DHS data we need to adjust for clustering design of the study. I wonder if the authors have considered the clustered design of DHS data in their analysis.

Result:

- I did not understand when the authors mentioned 68031 children under 36 months were included. Also, why children under 71 months? That is beyond 5 years. In the method section, the sample was children 3-5 years old.
- I do not think that the authors can come up with such an outcome interpretation that for every 1% increase in exposure to certain violence how much the prevalence of ECC increases as the p values are far from significant and i.e. the reported outcome is not associated to the exposure. Even if there is a trend there.

Discussion:

- I think the whole discussion is written in a sense that “there is an association between exposure to IPV and ECC” which is definitely not correct. Since there was no association found in the analysis, I encourage the authors to rewrite the discussion around that. In this format I think the discussion is misleading as it focuses on the reasoning why maternal experience of IPV is associated with ECC. Which is an explanation of non-significant findings.

Is the work clearly and accurately presented and does it cite the current literature?

Yes

If applicable, is the statistical analysis and its interpretation appropriate?

I cannot comment. A qualified statistician is required.

Are all the source data underlying the results available to ensure full reproducibility?

Yes

Is the study design appropriate and is the work technically sound?

Partly

Are the conclusions drawn adequately supported by the results?

No

Are sufficient details of methods and analysis provided to allow replication by others?

Partly

Reviewer Expertise:

Intimate partner violence_Maternal and child health_epidemiology

I confirm that I have read this submission and believe that I have an appropriate level of expertise to state that I do not consider it to be of an acceptable scientific standard, for reasons outlined above.

# Response

**Published:** 2021-11-08

**Authors:** Morenike Oluwatoyin Folayan

**Affiliations:** Obafemi Awolowo University, Nigeria

The paper discussed a very important issue and has a very interesting and important research question. However, I am concerned that the interpretation of the results is misleading, and the authors have gone a bit too far in trying to interpret the non-significant findings. The results as I can see are that there is no association between maternal experience of IPV and ECC. Which, as the authors mentioned, might be because of a small sample size. However, based on the p values and effects size the interpretation cannot go beyond. Please find some detailed comments below:

**Response:** Thanks for the positive feedback. We have addressed the comments. Your comments have helped to improve the quality of the manuscript. We hope the reviewers finds the revision has addressed the concerns raised

 Introduction:

- I believe the author can develop on the association between maternal experience of IPV and child health. While stunting is definitely one of the measured outcomes, there are several negative outcomes on children’s health which can be mentioned. Further, the pathways through which maternal experience of IPV can affect their children’s health can be discussed a bit more. The authors have a very strong approach by using social cognitive theory. As much as self-efficacy is an important and very well-known reason, there are other possible explanations linking maternal exposure to violence and their children’s health including dental health.
**Response:** We have improved on the introduction as suggested. Please see paragraph 1 and 2 of the introduction
- “Countries where data is available” is quite a vague description. I recommend that authors talk about the number of the countries, region and list of them.
**Response:** We have listed the 20 countries. Please see the third paragraph in the methodology section.
- Methods. More information regarding DHS and El tantawi study is required. The number of included and excluded studies is needed in the methods
**Response: **We have provided more details on the El tantawi study. Please see the paragraph 1 and 2 of the methodology section.

Statistical analysis: I am not an expert in ecological studies but I know in DHS data we need to adjust for clustering design of the study. I wonder if the authors have considered the clustered design of DHS data in their analysis.

**Response:** We agree with the Reviewer's comment. We used the weights in d005 of the DHS datasets for the values in table 2.  

 Result: I did not understand when the authors mentioned 68031 children under 36 months were included. Also, why children under 71 months? That is beyond 5 years. In the method section, the sample was children 3-5 years old.

**Response:** 71 months is 5 years.

  

- I do not think that the authors can come up with such an outcome interpretation that for every 1% increase in exposure to certain violence how much the prevalence of ECC increases as the p values are far from significant and i.e. the reported outcome is not associated to the exposure. Even if there is a trend there.
- **Response: **In agreement with the Reviewer's comment and to clarify our meaning and avoid misleading readers, we modified the results to reflect that the association was not significant. We based our interpretation on statistical convention ( https://www.wiley.com/WileyCDA/Section/id-831952.html AND https://home.csulb.edu/~msaintg/ppa696/696stsig.htm) that tests of significance have p values that show whether associations are significant or not. Therefore, associations can be significant or non-significant. Also, significance is related to sample size and for the same variables and associations, increasing the sample size may change a non-significant association into a significant association. We thus based our interpretation of the analysis on the direction and magnitude of the association and now explicitly indicated that it was non-significant.

Discussion: I think the whole discussion is written in a sense that “there is an association between exposure to IPV and ECC” which is definitely not correct. Since there was no association found in the analysis, I encourage the authors to rewrite the discussion around that. In this format I think the discussion is misleading as it focuses on the reasoning why maternal experience of IPV is associated with ECC. Which is an explanation of non-significant findings.

**Response:** Thanks for highlighting this limitation with the discussion. We have revised the discussion to highlights the non-significant relationship between IPV and ECC, and the implications of the findings. We hope the reviewer will find the revision a lot more representative of the study findings and suitable                             
